# Feasibility of establishing a biosafety level 3 tuberculosis culture laboratory of acceptable quality standards in a resource-limited setting: an experience from Uganda

**DOI:** 10.1186/1478-4505-13-4

**Published:** 2015-01-15

**Authors:** Willy Ssengooba, Sebastian J Gelderbloem, Gerald Mboowa, Anne Wajja, Carolyn Namaganda, Philippa Musoke, Harriet Mayanja-Kizza, Moses Lutaakome Joloba

**Affiliations:** Department of Medical Microbiology, Makerere University College of Health Sciences, School of Biomedical Sciences, P.O. Box 7072, Kampala, Uganda; Department of Global Health and Amsterdam Institute of Global Health and Development, Academic Medical Center, University of Amsterdam, P.O. Box 22700, 1100 DE Amsterdam, Netherlands; Aeras Global TB Vaccine Foundation, South Africa, Black River Park, Observatory, Cape Town, 7925 South Africa; Makerere University College of Health Sciences, Infectious Diseases Institute, P.O. Box 22418, Kampala, Uganda; Makerere University Johns Hopkins University Research Collaboration, P.O. Box 23491, Kampala, Uganda; Makerere University College of Health Sciences, School of Medicine Kampala Uganda, P.O. Box 7072, Kampala, Uganda

**Keywords:** Acceptable quality standards, Biosafety level 3, Feasibility, Resource limited countries, TB culture

## Abstract

**Background:**

Despite the recent innovations in tuberculosis (TB) and multi-drug resistant TB (MDR-TB) diagnosis, culture remains vital for difficult-to-diagnose patients, baseline and end-point determination for novel vaccines and drug trials. Herein, we share our experience of establishing a BSL-3 culture facility in Uganda as well as 3-years performance indicators and post-TB vaccine trials (pioneer) and funding experience of sustaining such a facility.

**Methods:**

Between September 2008 and April 2009, the laboratory was set-up with financial support from external partners. After an initial procedure validation phase in parallel with the National TB Reference Laboratory (NTRL) and legal approvals, the laboratory registered for external quality assessment (EQA) from the NTRL, WHO, National Health Laboratories Services (NHLS), and the College of American Pathologists (CAP). The laboratory also instituted a functional quality management system (QMS). Pioneer funding ended in 2012 and the laboratory remained in self-sustainability mode.

**Results:**

The laboratory achieved internationally acceptable standards in both structural and biosafety requirements. Of the 14 patient samples analyzed in the procedural validation phase, agreement for all tests with NTRL was 90% (*P* <0.01). It started full operations in October 2009 performing smear microscopy, culture, identification, and drug susceptibility testing (DST). The annual culture workload was 7,636, 10,242, and 2,712 inoculations for the years 2010, 2011, and 2012, respectively. Other performance indicators of TB culture laboratories were also monitored. Scores from EQA panels included smear microscopy >80% in all years from NTRL, CAP, and NHLS, and culture was 100% for CAP panels and above regional average scores for all years with NHLS. Quarterly DST scores from WHO-EQA ranged from 78% to 100% in 2010, 80% to 100% in 2011, and 90 to 100% in 2012.

**Conclusions:**

From our experience, it is feasible to set-up a BSL-3 TB culture laboratory with acceptable quality performance standards in resource-limited countries. With the demonstrated quality of work, the laboratory attracted more research groups and post-pioneer funding, which helped to ensure sustainability. The high skilled experts in this research laboratory also continue to provide an excellent resource for the needed national discussion of the laboratory and quality management systems.

**Electronic supplementary material:**

The online version of this article (doi:10.1186/1478-4505-13-4) contains supplementary material, which is available to authorized users.

## Background

Tuberculosis (TB) remains a global emergency especially in resource-limited settings [1]. Laboratory diagnosis is vital for the control of pulmonary TB. Even with available new diagnostics, culture remains the most sensitive diagnostic method, especially among HIV-infected patients and infants as well as for novel TB vaccines and drugs trials for baseline and end-point determination [2, 3].

The emergence of multidrug resistant (MDR) and extensively drug resistant TB has increased the need to establish more facilities for culture-based drug susceptibility testing (DST), at least until better molecular tests that offer a complete set of DST results needed for patient management become available. Recently, the World Health Organization (WHO) endorsed the use of the Xpert MTB/RIF assay (Cepheid, Sunnyvale, CA, USA) as a major tool for rapid and sensitive detection of rifampicin resistance [2, 4]. However, Xpert rifampicin resistant results need to be carefully interpreted with consideration of the risk of MDR-TB in a given patient and the expected prevalence of MDR-TB in a given setting [4]. Also, additional drug resistance profiles are required for management and treatment monitoring of MDR-TB patients for which currently no alternatives to culture-based DST have been endorsed.

The WHO biosafety guidelines stipulate that any manipulation of samples suspected of containing MDR *M. tuberculosis* should be conducted in a biosafety level 3 (BSL-3) facility [5]. Culture for TB is routine in most high-income countries, however, with the initial and operational requirements associated with this type of laboratory, it may not be affordable by most low-income countries that are highly burdened with TB [6–8]. Therefore, for middle- and low-income countries, the WHO recommends a stepwise approach with regards to the introduction of culture and DST, especially on liquid media systems [5].

Uganda, with a population of 34,509,000 people, has an approximate TB incidence of 193/100,000 population/year with an estimated TB incidence among the HIV-infected of 53/100,000 population/year [1]. In 2012, the prevalence of MDR-TB was 1.2% and 12% among new and retreatment cases, respectively [1, 9]. There is only one public TB culture laboratory in Uganda, although there are, in addition, four clinical research TB culture laboratories that do not participate in routine patient care. Sharing experiences in the setting-up of a BSL-3 laboratory and other operational issues may guide the decentralization processes to the areas where TB culture services can easily be accessible to TB patients, especially the HIV-infected and MDR-TB suspects. It can also help in realizing the research and development agenda towards TB control. Herein, we share our experience of establishing a BSL-3 culture facility in Uganda as well as 3-years performance indicators and post-TB vaccine trials (pioneer) and funding experience of sustaining such a facility.

### Legal and operational framework of TB laboratories in Uganda

The objectives of constructing the current laboratory at Makerere University Teaching Hospital were to participate in TB-related clinical research, training health care workers, and to work in collaboration with the National TB Reference Laboratory (NTRL) towards TB control through sharing evidence-based data and information.

According to the Uganda National Health Laboratory Services Policy in 2009, at the national level, the NTRL and the Central Public Health Laboratories oversee the activities of all public TB laboratories, which currently only perform sputum smear microscopy [10]. The current laboratory is under Makerere University’s National Teaching and Referral Hospital, so it is legally under the Ministry of Education and Sports but also works closely with the Ministry of Health through training health workers. It existed on the ground of bridging the gap between operational and clinical or biomedical research, as it was a big task for the NTRL/Central Public Health Laboratories to conduct both types of research fully, in addition to patient care, supervision of other laboratories, and conducting operational/implementation research.

### Biosafety level 3 (BSL-3) laboratory

A BSL-3 laboratory is a containment facility that enables the isolation and manipulation of organisms belonging to risk group three of infectious organisms. These organisms are categorized as having high individual risk and low community risk. The pathogens in this class usually cause serious human and animal disease and do not ordinarily spread from an infected individual to another. For risk group three organisms, effective treatment and preventative measures are available [11]. Standards for BSL-3 allow manipulation of pathogens that can be transmitted through aerosols. Organisms that are more risky than level three, i.e., risk group four, such as those causing Ebola, are handled in maximum containment laboratories with biosafety level four standards [7, 11]. While set-up and maintenance of such facilities is less affordable in most low-income countries, the steady increase in complex public health problems and the outbreak of serious disease is likely to lead to increased demand for such facilities.

## Methods

### Establishing the laboratory: from the first stone to operational approval

#### Laboratory design and construction

Between September 2008 and April 2009 a BSL-3 TB culture laboratory measuring 4.5 meters by 6.5 meters wide was constructed in Makerere University College of Health Sciences, Department of Medical Microbiology. We considered building the structure instead of buying a modular laboratory because we thought that a modular design may not be easy to sustain and durable given the challenges in our setting in terms of environment, electricity instability, and lack of technical expertise in case of system breakdown.

It was set to participate in the planned Phase III TB vaccine trials among infants and adolescents. The facility was constructed and equipped with financial support from Aeras Global TB Vaccine Foundation. Makerere University offered in-kind contribution in terms of space for construction, an architectural consultant, water, and electricity, and facilitated the laboratory’s legalization. Following Ministry of Public Services guidelines, a laboratory manager was recruited to oversee the construction, follow-up on agreed upon construction milestones, and operation of the proposed TB culture laboratory. A local architectural firm was identified for the design using the WHO Laboratory Biosafety Manual 2004 [11] as a reference guide for specifications. The Uganda Ministry of Health guidelines for establishing a laboratory as well as Ministry of Public Services guidelines for identifying local constructors were followed throughout this process. Biosafety standards from WHO, 2004 [11], were used as a reference guide by an independent firm of engineers and architects to verify compliance with the WHO laboratory biosafety design and facilities requirements.

#### Laboratory equipment and human resources

Funders were requested to purchase the identified equipment directly from the suppliers following their national guidelines. The cost for laboratory construction in the year 2009 was approximately US$ 130,000. Start-up equipment included two incubators, a –80°C and a –20°C freezer, four refrigerators, three class II biosafety cabinets, a centrifuge, two fluorescence microscopes, a halogen Olympus BX51 and a light-emitting diode (LED) Olympus CX31 (Fraen Corporation S.r.l.-USA), a weighing balance, a pH meter, an autoclave, a 100 KVA back-up generator, a central UPS and power stabilizer, four carbon dioxide gas supplies, two mycobacterial growth indicator tube (MGIT) machines (Becton and Dickson, Franklin Lakes, NJ, USA), and an ultrasonic cleaning bath, as well as other supplies and equipment. The equipment cost amounted to US$ 242,324, and the apparatus and glassware costs to US$ 22,496. Start-up consumables and reagents for one year cost US$ 134,655. The partners supported the laboratory with full operational costs, including salaries, additional reagents, and supplies, continuously for their specified study period. It was anticipated that at the end of the planned study period, the laboratory would be fully supported by Makerere University and its collaborators for sustainability. The studies ended in 2011 and the laboratory currently depends on contributions from other research projects in terms of user fees.

During and after its construction, the laboratory received technical support from partners to ensure that the code of practice as well as health and medical surveillance requirements for a BSL-3 laboratory, as specified by the WHO biosafety guidelines, were realized. Additional files 1,2,3 and 4 show the main operational areas of the BSL-3 laboratory. More support to register for external quality assessment (EQA) from partners as part of preparedness activities for participation in the planned Phase III TB vaccine trials among infants and adolescents and international accreditation was also offered. On the 15^th^ of September 2009, the laboratory was approved by the Ministry of Health of Uganda to operate as a TB culture facility and also fulfilled the WHO requirements for a BSL-3 laboratory classification [11]. Thereafter, the lab manager received a further one-month training in managing a BSL-3 facility from St. Johns Academy of Health Sciences Institute of Infectious Diseases, Bangalore, India. Later two graduate laboratory technologists and one graduate data administrator were recruited and the laboratory manager and the laboratory supervisor, who had prior experience in TB culture procedures, trained them. All personnel were recruited following the Uganda Ministry of Public Services guidelines and fully paid by funders of the TB vaccine trial through the study period. In order to save time for the planned studies to begin and not to “reinvent the wheel”, standard operating procedures (SOPs) were adopted from St. Johns Academy and from the NTRL and customized to the current laboratory setting in reference to standard smear microscopy and culture procedures [12, 13]. Laboratory activities: from Validation phase to full force testing.

#### Validation phase

Once all equipment were in place, validation activities for the newly customized SOPs and reliability of the new equipment were conducted. This was performed using routine sputum samples from routine TB patients attending Mulago National Referral Hospital, which were tested in parallel with the NTRL from March to June 2009. Samples from routine TB patients were used to reduce delays that would have been required due to approvals and consenting processes if patients had been approached. Each sample was parallel tested using LED fluorescence smear microscopy (Ziehl-Neelsen was not done), culture using home-made Lowenstein-Jensen (LJ) media, commercial MGIT and identification for MGIT using Capillia Neo TB™ (TAUN, Numazu, Japan), and the laboratory used morphological identification for colonies observed on LJ in accordance with the same SOPs. To test the SOPs, technical competency, and the equipment used for DST, 10 American Type Culture Collection strains of H_37_RV with known susceptibility results were used aiming at 100% reproducibility.

A validation phase was planned with the NTRL, which had similar equipment and procedures. The reference laboratory, NTRL, was selected due to the fact that it had a registered decade-long record of competence in these tests and it is currently a supra-national TB reference laboratory with ISO15189 accreditation [14]. Results from NTRL were considered as the reference results and the acceptable level of agreement and reproducibility to start analyzing patient’s sputum samples was set at 80% and above for all methods. This was also adopted for all EQA programs thereafter. As planned, corrective and preventive action projects for discordant results were performed.

The laboratory started full operations in October 2009 performing fluorescence smear microscopy, culture, identification, and DST according to internationally accepted safety, technical and quality standards for TB culture [15, 16], and in the same period registered for EQA panels. The panels were from the WHO, the National Health Laboratory Services (NHLS), and the College of American Pathologists (CAP). All smear microscopy examination was performed by the auramine-O-phenol fluorescence method. Laboratory performance was assessed using the standard performance indicators for mycobacteriology laboratories. Early and continuous corrective and preventative projects were implemented for non-compliances.

### Ethical considerations

This study was nested in the main TB vaccine preparatory study that was approved by the Makerere University School of Public Health–Higher Degrees and Research Ethics committee (HDREC), Kampala Uganda and the Uganda National Council of Science and Technology (UNCST).

## Results

### Laboratory design and biosafety

The laboratory met the structural and biosafety requirements as stipulated by the WHO Laboratory Biosafety Manual, 3^rd^ edition, for BSL-3 laboratories [11]. Tables 1 and 2 and Additional files 1,2,3 and 4 show the items as expected and achieved.

**Table 1:** **Laboratory design and main equipment in BSL-3 laboratory according to WHO biosafety guidelines**

Item	Biosafety level 3		
	WHO biosafety manual	Current laboratory	Description
Isolation^a^ of laboratory	Yes	Yes	On the topmost level of the building with highly restricted access
Room sealable for decontamination	Yes	Yes	All windows are sealed and checked for leakages
Ventilation			
Inward airflow	Yes	Yes	Inward airflow and ventilation system is automatically monitored with auto-sensors and two Magnehelic® gauges (for supply and exhaust each) and re-validated and certified annually by Air-filter maintenance services (AFMS) group of South Africa
Controlled ventilating system	Yes		
HEPA-filtered air exhaust	Yes/No^b^		
Double-door entry	Yes	Yes	Doors are well sealed
Airlock	No	No	
Airlock with shower	No	No	
Anteroom	Yes	Yes	Anteroom is under negative pressure
Anteroom with shower	Yes/No^c^	No	
Effluent treatment	Yes/No^c^	No	All effluent is decontaminated in the BSL-3 laboratory before is discharged to join the sewage system of the National Referral Hospital (Mulago)
Autoclave			Inside the containment section of the laboratory
On site	Yes	Yes	
In laboratory room	Desirable	Yes	
Double-ended	Desirable	No	
Biological safety cabinets	Yes	Yes	Well serviced by AFMS annually
Personnel safety monitoring capability^d^	Desirable	Yes	Reception and the laboratory sections are connected to intercom system and CCTV camera with a link to the laboratory manager and director’s offices

^a^Environmental and functional isolation from general traffic.

^b^Dependent on location of exhaust.

^c^Dependent on agent(s) used in the laboratory.

^d^For example, window, closed-circuit television, two-way communication.

**Table 2:** **Containment laboratory – biosafety level 3: laboratory safety survey checklist adopted from WHO biosafety manual, 3** ^**rd**^ **edition** [11]

Item	Achieved	Frequency/description
**Personal protection**	Yes	There are two sets of gowns clearly labelled to differentiate those strictly used in containment section
Closed-front gowns worn in laboratory		
Protective laboratory clothing worn only in laboratory areas	Yes	All section have their respective clothing readily available in areas of use
Hand-washing sink foot, elbow or automatically controlled	Yes	With elbow operated tapes
**Hand protection**	Yes	All areas where infectious materials are handled have sign post for use of gloves as a reminder
Double gloves worn when handling infectious material, potentially contaminated equipment and work surfaces		
**Respiratory protection**	Yes	All samples are checked for leakages before accessioning and processed inside a class II biosafety cabinet
Respiratory protection worn by all personnel in the laboratory when aerosols are not safely contained in a biosafety cabinet (BSC)		
**Practices**	Yes	N95 masks, gaggles, and head-dress are available and strictly used when handling chemicals and materials that can affect mucous membrane
Mucous membrane protection provided when working with infectious material outside a BSC		
Personnel advised of special hazards associated with the agent(s)	Yes	Through biosafety manual and initial and refresher trainings. All SOPs have a safety precaution section
Personnel required to read and follow all instructions on practices and procedures, including safety or operations manual	Yes	This is quarterly checked through internal audits and safety audits
Personnel receive annual updates/additional training for procedural changes	Yes	Through in-house training facilitated by biosafety officer or external trainings; also key points are emphasized during biosafety meetings
All contaminated waste autoclaved prior to disposal	Yes	Using disinfectants and autoclave before disposal

### Laboratory performance in the validation phase

Only 14 randomly selected patient samples were analyzed during the validation phase due to the limited time that was allocated for this exercise before the studies could begin. All samples tested in parallel with NTRL for smear microscopy and culture on both LJ and MGIT had kappa values of 0.9 (*P* <0.01; Table 3). The DST results had 100% reproducibility scores for the drugs tested, namely streptomycin, isoniazid, rifampicin, and ethambutol (data not shown).

**Table 3:** **Validation results by parallel testing with the National TB Reference Laboratory (NTRL) as reference (n = 14)**

Current laboratory	NTRL			
Test method	Results	Positive	Negative	Total
**Smear microscopy**	Positive	7	0	7
	Negative	1	6	7
	Sensitivity			87.5
	Specificity			100
	Kappa			0.9
**Sputum culture (MGIT)**	Positive (MTBc)	7	0	7
	Negative	1	6	7
	Sensitivity			87.5
	Specificity			100
	Kappa			0.9
**Sputum culture (LJ)**	Positive (MTBc)	6	0	6
	Negative	1	7	8
	Sensitivity			85.7
	Specificity			100
	Kappa			0.9

LJ, Lowenstein-Jensen; MGIT, Mycobacterial growth indicator tube; MTBc, Mycobacterium tuberculosis complex.

### Laboratory performance indicators (2010 to 2012)

Standard performance indicators for TB culture laboratories [17, 18] were also calculated and interpreted on a weekly, monthly, and quarterly basis for continuous quality improvement projects. Here, results from January 2010 to December 2012 are presented.

From January 2010 to December 2012, the laboratory performed 20,590 cultures on both MGIT and LJ methods, of which 37.1% (7,636) were for 2010, 49.7% (10,242) were for 2011, and 13.2% (2,712) were for 2012. Of these 20,590 cultures, 50.9% (10,490) were performed on LJ and 49.1% (10,100) on MGIT. All samples were from clinical research studies.

In the years 2010, 2011, and 2012, the contamination rates for LJ culture were 1.8% (70/3,818), 5.5% (287/5,186), and 3.2% (48/1,486), whereas contamination rates for MGIT cultures were 8.2% (314/3,818), 26.1% (1,321/5,056), and 11.2% (137/1,226), respectively (Table 4). These percentages were compared to internationally-accepted standards (2% to 5% for LJ and 6% to 10% for MGIT), and corrective and preventive action projects were initiated for non-conformity. The mycobacterium tuberculosis complex (MTBc) positivity rates by LJ cultures were 1.8% (69/3,818), 13.2% (683/5,186), and 30.1% (447/1,486), whereas for MGIT cultures these were 2.8% (107/3,818), 15.4% (777/5,056), and 28.7% (352/1,226) for the years 2010, 2011, and 2012, respectively. There was an increase in the number of smear-positive but culture-negative patients from 0.6% to 6.9% on LJ and 0.2% to 1.7% on MGIT between the years 2010 and 2012. Table 4 summarizes the performance for culture methods in relation to smear microscopy.

**Table 4:** **Performance of culture in relation to smear microscopy (2010–2012)**

Culture method	Performance indicator	2010 N (%)	2011 N (%)	2012 N (%)
**LJ culture**	**Total (n)**	**3,818**	**5,186**	**1,486**
	Culture positive (MTBc)	69 (1.8)	683 (13.2)	447 (30.1)
	Culture negative	3,749 (98.2)	4,503 (86.8)	1,039 (69.9)
	Culture contaminated	70 (1.8)	287 (5.5)	48 (3.2)
	Smear negative among culture positive	19 (27.54)	255 (37.3)	172 (38.5)
	Smear positive among culture positive	46 (66.7)	377 (55.2)	264 (59.1)
	Smear positive among culture contaminated	3 (4.3)	54 (18.8)	14 (29.2)
	Smear positive among culture negative	21 (0.6)	66 (1.5)	72 (6.9)
**MGIT culture**	**Total (n)**	**3,818**	**5,056**	**1,226**
	Culture positive *M. tuberculosis* (MTBc)	107(2.8)	777 (15.4)	352 (28.7)
	Culture negative	3,285 (86.0)	2,705 (53.5)	682 (55.6)
	Culture positive with MOTT	112 (2.9)	253 (5.0)	55 (4.5)
	Culture contaminated	314 (8.2)	1,321 (26.1)	137 (11.2)
	MOTT among smear positive	2 (2.8)	33 (6.6)	14 (6.6)
	Smear negative among culture positive (MTBc)	56 (52.3)	575 (74.0)	211 (60.0)
	Smear positive among culture positive (MTBc)	62 (57.9)	452 (58.2)	195 (55.4)
	Smear positive among culture contaminated	0 (0.0)	23 (1.7)	5 (3.6)
	Smear positive among culture negative	8 (0.2)	23 (0.8)	12 (1.7)

Mycobacterial identifications on LJ were done basing on morphological features and Ziehl-Neelsen microscopy were done on doubtful colonies [15].

LJ, Lowenstein-Jensen; MGIT, Mycobacterial growth indicator tube; MOTT, Mycobacterium other than tuberculosis; MTBc, Mycobacterium tuberculosis complex.

In December 2009, the laboratory registered to participate in quarterly EQA procedures with the NHLS, South Africa, and through the NTRL, in the WHO DST EQA panel from the Supranational TB Reference Laboratory at the Institute of Tropical Medicine, Antwerp, Belgium. In the fourth quarter of 2011, the laboratory was registered for CAP EQA panels. All these EQA procedures checked the available standard tests for patient management.

### Laboratory performance in EQA panels from 2010 to 2012

**Figure 1:**
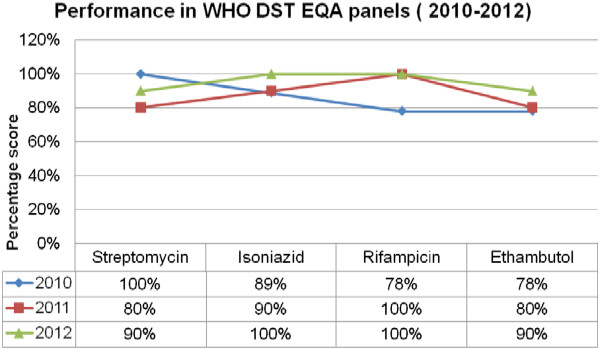
**Performance in WHO drug susceptibility testing DST and External quality assessment EQA panels (2010–-2012).** DST = Drug Susceptibility Testing, EQA = External.

**Figure 2:**
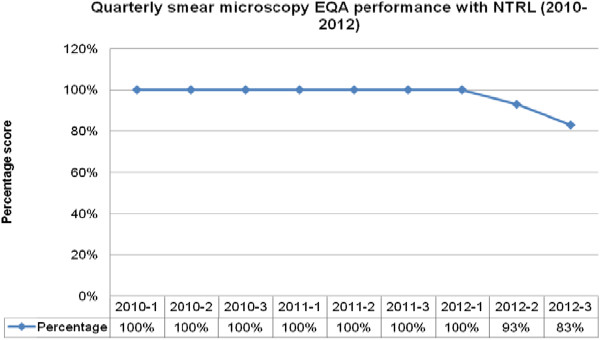
**Quarterly smear microscopy External quality assessment EQA performance with the NTRL National Tuberculosis Reference Laboratory (2010–-2012).** EQA = External , NTRL = National Tuberculosis Reference Laboratory.

The laboratory performed with a reproducibility of 80% and above throughout the years of participation in the WHO DST EQA panels for all drugs except for 2010 on rifampicin and ethambutol. There was good to fair baseline performance in the year 2010 with an increase in scores in 2011 and 2012 (Figure 1). For the quarterly smear microscopy EQA panels with NTRL [3], there was exceptional performance of 100% across quarters of the years with slight reduction in scores in the year 2012 (Figure 2).

**Figure 3:**
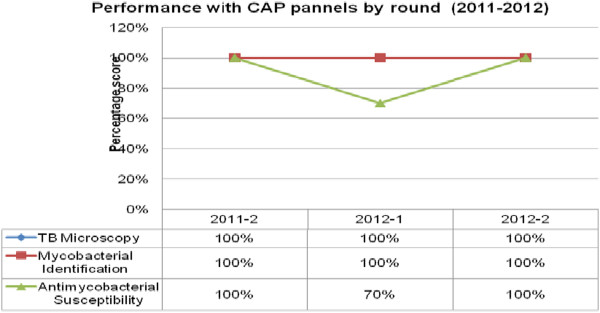
**Performance with College of American Pathologists’CAP panels by rounds (2011–-2012).** CAP = College of American Pathologists.

**Figure 4:**
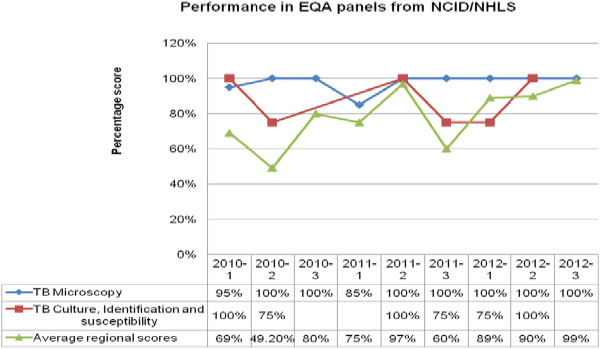
**Performance in external quality assessment EQA panels from NCID/NHLS.** NCID/NHLS = the National Institute for Communicable Diseases and the National Health Laboratory Service.

With the CAP EQA panels, the laboratory scored 100% for smear microscopy and sputum culture across the participation period, with a reduction in scores to 70% for antimycobacterial susceptibility/DST in one quarter (Figure 3). For the NHLS EQA panel participation for smear microscopy, culture, identification, and susceptibility, the laboratory had between acceptable and fair scores (80% and above and 75% and above, respectively) in all rounds of participation (Figure 4).

## Discussion

This experience from Uganda indicates that, with collaborative efforts, funding, and technical support from locally available expertise, it is feasible to set-up and optimally operate a BSL-3 TB culture and DST facility with acceptable quality performance in a resource-limited setting. Knowing the national and international requirements for construction and equipment procurement as well as personnel recruitment prior to initial laboratory set-up is key to achieving the set start-up targets. Initial and continuous support from the partners as well as open discussions on the possible limitations and finding out the alternative solutions ahead of time is also crucial.

Understanding the acceptable performance standards as well as planning the routes to achieving them upfront, including early monitoring of performance with corrective and preventative action projects, is important if standards are to be realized. From our experience, this requires the laboratory to have passed the validation phase with sensitivity scores of at least 80% as well as early monitoring of performance indicators for a TB culture laboratory.

From our findings, culture contamination rates, though mainly within the acceptable ranges, may potentially increase with increasing workload. The majority of specimens processed in 2011 were from the adolescent study collected unsupervised from their homes of a distant field site to the processing laboratory. High contamination rates from unsupervised samples have also been documented in previous studies [19, 20].

During this period, there was an increase in positivity rates over the years and this was attributed to the type of the studies that were conducted at the laboratory per year. The laboratory mostly processed samples from infant and adolescent cohort studies from the TB vaccine preparedness studies seeking to establish baseline epidemiological indices for the planned TB vaccine trials between 2010 and 2011, and adult studies from mainly HIV-infected TB suspects including TB patients added on from 2011 to 2012.

There was high mycobacterium other than tuberculosis recovery with MGIT culture which also increased with increasing workload. This is because MGIT has more nutrients that support recovery of the fast growers [21, 22]. The recovery was high in the year 2011 as this was the peak for the adolescent cohort study, which comprised of about 80% of the laboratory workload that year. The mycobacterium other than tuberculosis rate in adolescents is consistent with the previous study among adolescents [23].

There was an increase in the number of smear-positive but culture-negative patients (Table 4) over the years due to inclusion of studies that had TB patients who were on treatment for treatment monitoring/outcome studies from mid-2011 through 2012. Previous studies have shown that patients who are on treatment for monitoring purposes and smear positive are likely to be culture negative [24–26].

There were exceptional scores from quarterly EQA performance of smear microscopy with NTRL in 2010 and 2011, with a slight decline in two quarters of 2012 (Figure 2). The decline was attributed to the period of learning curve for the technologists before their competency was stabilized. This mainly led to low false negative smear readings.

Culture scores were 100% for CAP panels (Figure 3) and were within acceptable range (majority 80% and above) in all procedures for all years for WHO (Figure 1) and fair performance (majority 75 and above) for NHLS (Figure 4). This was possible due to the strict proficiency procedures in the laboratory, which require one senior and one junior technologist in the section every quarter to ensure effective competency transfer to the junior technologist. The scores in the DST for WHO and CAP were mainly attributable to competency gain across the years and an oversight in monitoring in the laboratory’s cold storage system that could have led to loss of potency from the streptomycin drug that was accidently used in the panels of 2011. The low performance in 2010 were mainly attributed to competency gain phase. All performances below 100% were investigated and corrective and preventative actions established.

However, our findings had some limitations. First, failure to use biochemical tests for identification of MTBc on LJ, instead of morphological identification methods, may have led to misidentification errors that can lead to over-estimation of MTBc recovery on LJ. However, this may not have been the case with our findings as previous studies in Uganda have indicated less need for non-morphological methods when using LJ since all growth from those studies on LJ was for MTBc [20, 21]. Secondly, the number of samples tested during the validation period could have been small for a conclusive evidence of personnel competency and validation. However, the subsequent evidence of quality performance overrules that concern. Furthermore, the percentage of smear-positive but culture-negative results may have suffered an aggregation effect; disaggregating the figures to indicate the number of those who were on TB treatment in a given period and those had never been on TB treatment may show a different rate. It is also worth noting that EQA performance may not always translate to total quality assurance since acceptable internal measures are required to reduce performance gaps.

### Threats and opportunities towards sustainability

After the end of support from the main studies that led to construction of this facility, the pioneer funders offered one year transitional support and the laboratory administration was returned to Makerere University. There were challenges of maintaining the laboratory, including the servicing of key equipment and ventilation system, and non-permanent university staff salaries. However, operational costs and salaries of the key leadership team members (permanent university staff) were met by the Makerere University. The laboratory leadership, through Makerere University, played a big part to alert possible collaborators of the available opportunity for collaboration. The good reports of continuous acceptable laboratory performance from international auditors and monitors, as proof of quality and reliability, were very instrumental towards attracting more collaborators and studies. With the demonstrated quality of work and acceptable standards achieved up to that time, the laboratory attracted more research groups and studies following the pioneer funding. The cost per test was determined for all tests/services offered based on the cost of reagents and other consumables as well as the amount of time required to perform the test, and the fees generated are used to support salaries and the maintenance of infrastructure and equipment (biosafety cabinets, incubators, generator ventilation system, etc.). There is still a challenge of maintaining the established safety standards and practices in terms of continuous acceptability and costs. Furthermore, adequate biosafety in BSL-3 can mainly be ensured if all concepts are understood and acted upon and it is a continuous learning experience [7, 27]. Continuous evaluations and performance improvement projects performed by QMS and safety officers as well as increased commitment of the laboratory staff towards achieving acceptable standards are likely to lessen this threat. There are variations in minimum safe BSL-3 practices across global regions of collaborators which are sometimes contradictory and still pose a challenge, for example, the recommendation or not of the use of a mask in the containment section, among others [7, 27, 28].

## Conclusions

It is feasible to establish a BSL-3 TB culture laboratory with acceptable quality performance standards in resource-limited countries. Early and continuous monitoring of performance indicators and corrective actions in all newly established TB culture facilities is recommended. The laboratory is currently reliant on research grants for sustainability. Although efforts are being made to attract more support from private hospitals and clinics, the support so far received from these avenues is very low compared to the expected. There is a greater need of government support to ensure sustainability of this establishment, which will contribute to the efforts of TB control through quality research and development services as well as to human resources through the provision of experts who can contribute to the improvement of health systems nationally and globally. Future exploration or assessments of the satisfaction of the laboratory worker with the BSL-3 laboratory practices and/or researchers or academia regarding the services offered by this facility in accordance with its mission are needed.

### Policy implications

In line with the Maputo Declaration of 2008 and with the Uganda National Laboratory Policy of 2009, the establishment of this laboratory is a strong pillar towards improving and sustaining access to quality laboratory services. Despite a clear recognition of the need for research and development toward TB control nationally and globally, there is little national budget support to research laboratories to address human resources, consumables, equipment servicing and maintenance, and training needs.

This facility has increased the capacity to develop and validate more effective ways to diagnose TB, and it provides unique training opportunities in TB diagnosis and clinical research. This laboratory is recognized by the WHO Stop TB Partnership as a regional training center for non-commercial DST methods, registered for CAP accreditation, approved for AIDS Clinical Trials Group, International Maternal Pediatric Adolescent AIDS Clinical Trials Group (IMPAACT), and the Global TB Drug Development Alliance (TB alliance), as well as by Tuberculosis Clinical and Diagnostic Research Consortium (TBCDRC) studies. The high skilled experts in this research laboratory also continue to provide an excellent resource for the needed of national discussion of the laboratory system, participate in quality management systems, and provide a forum for open discussions between service delivery and research laboratories in line with the Maputo Declaration of 2008. The Ugandan government must not only recognize the need but also support the sustainability of this research facility as part of the greater health system.

## Electronic supplementary material

Additional file 1: **Sample reception area of the biosafety level 3 laboratory.** (PDF 114 KB)

Additional file 2: **Pre-sample processing area of the biosafety level 3 laboratory.** (JPEG 82 KB)

Additional file 3: **Anteroom to biosafety level 3 laboratory.** (JPEG 54 KB)

Additional file 4: **Containment section of the biosafety level 3 laboratory.** (JPEG 79 KB)

## Abbreviations

BSL-3: Biosafety Level 3
CAP: College of American Pathologists
DST: Drug susceptibility testing
EQA: External quality assessment
LJ: Lowenstein-Jensen
MDR: Multidrug resistant
MGIT: Mycobacterial growth indicator tube
MTBc: Mycobacterium tuberculosis complex
NHLS: National Health Laboratory Services
NTRL: National Tuberculosis Reference Laboratory
SOPs: Standard operating procedures
TB: Tuberculosis
WHO: World Health Organization.
