# Supplementary figures and images for: Feasibility of establishing a biosafety level 3 tuberculosis culture laboratory of acceptable quality standards in a resource-limited setting: an experience from Uganda
Source: Health Res Policy Syst. 2015 Jan 15;13:4. doi: 10.1186/1478-4505-13-4 (PMC4326287; doi:10.1186/1478-4505-13-4)

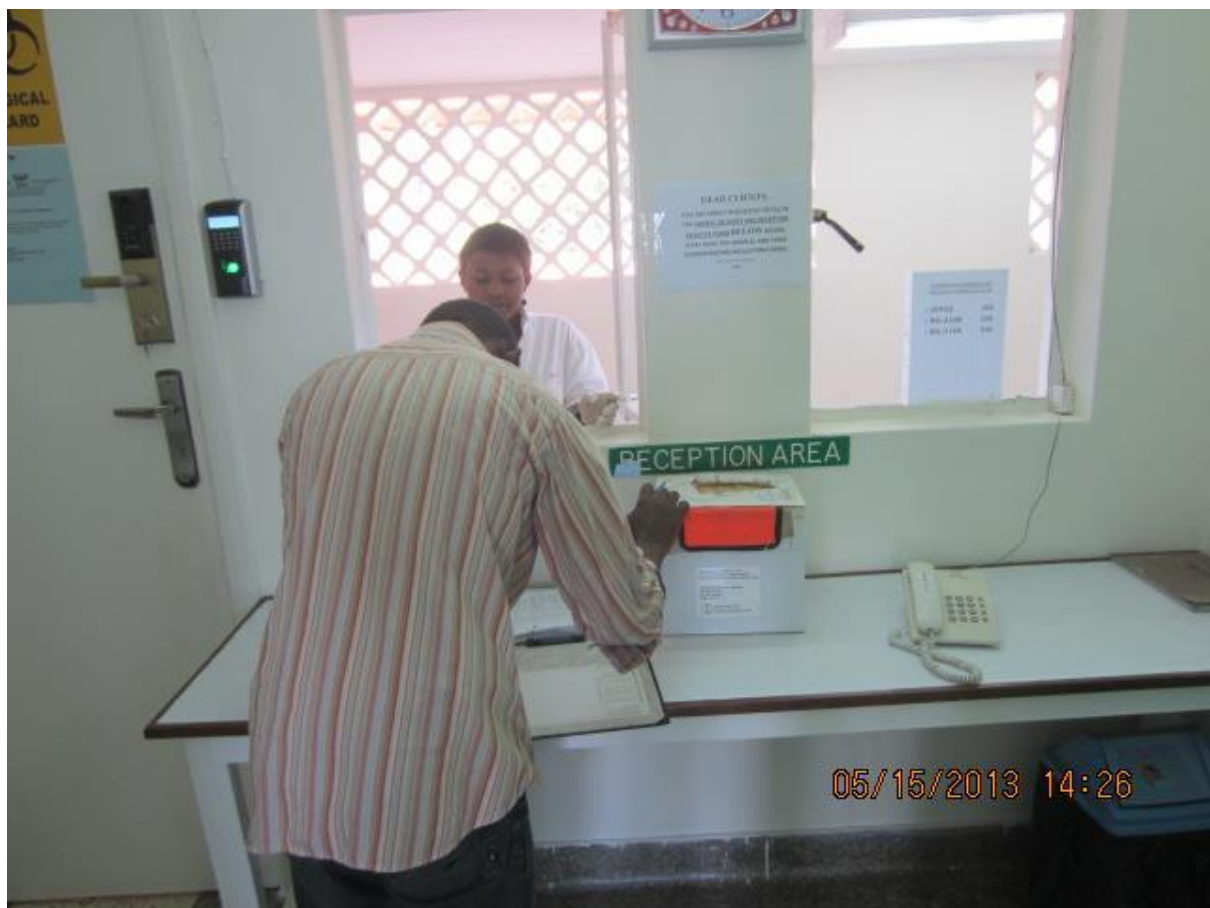

Supplement: Supplementary file 1 — Additional file 1: Sample reception area of the biosafety level 3 laboratory. (PDF 114 KB) [file 12961_2014_370_MOESM1_ESM.pdf]

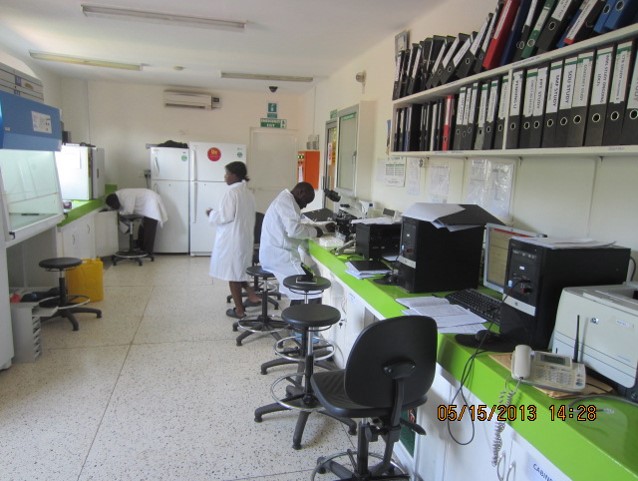

Supplement: Supplementary file 2 — Additional file 2: Pre-sample processing area of the biosafety level 3 laboratory. (JPEG 82 KB) [file 12961_2014_370_MOESM2_ESM.jpeg]

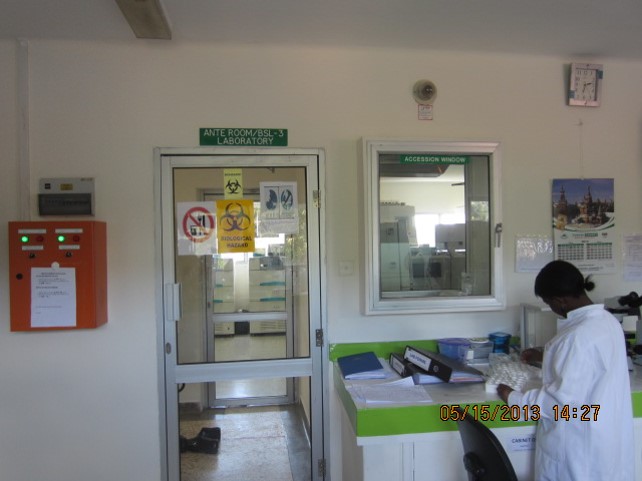

Supplement: Supplementary file 3 — Additional file 3: Anteroom to biosafety level 3 laboratory. (JPEG 54 KB) [file 12961_2014_370_MOESM3_ESM.jpeg]

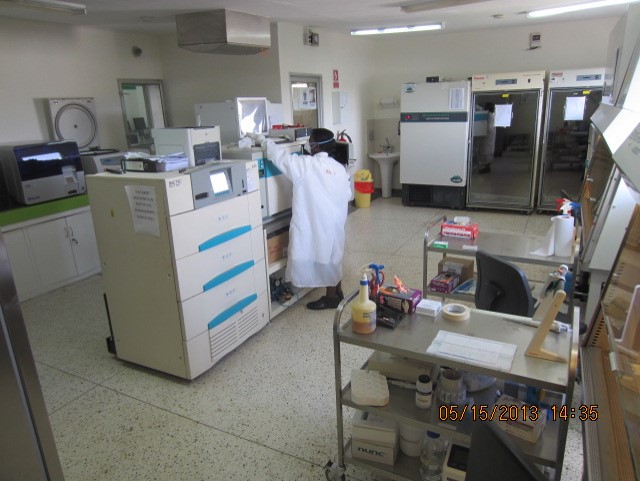

Supplement: Supplementary file 4 — Additional file 4: Containment section of the biosafety level 3 laboratory. (JPEG 79 KB) [file 12961_2014_370_MOESM4_ESM.jpeg]
